# The “angiogenesis-plaque stability paradox” in atherosclerosis pathogenesis

**DOI:** 10.3389/fcvm.2025.1659006

**Published:** 2025-10-02

**Authors:** Fei Yan, Si-yang Sun, Hong Wu

**Affiliations:** The Second Clinical Medical College, Henan University of Chinese Medicine, Zhengzhou, China

**Keywords:** atherosclerosis, angiogenesis, plaque stability, glycolysis, mast cells

## Abstract

Intraplaque angiogenesis, a critical mechanism in the pathological progression of atherosclerosis (AS), exhibits a paradoxical role by providing nutrients and repair support for plaques while simultaneously contributing to plaque instability and rupture. Current research on intraplaque angiogenesis primarily focuses on molecular mechanisms, cellular interactions, and metabolic regulation; however, its dual effects on plaque stability remain underexplored. This review elucidates the mechanisms underlying the angiogenesis-plaque stability paradox, including the glycolysis-lactate-lactylation modification axis, mast cell-mediated inflammatory responses, and angiogenic maturation and stabilization mechanisms, and discusses their roles and associated regulatory pathways in AS pathogenesis. These insights aim to potentiate atherosclerotic plaque stabilization and refine predictive accuracy for acute cardiovascular events.

## 1 Introduction

Atherosclerosis (AS) is a critical pathological foundation for cardiovascular diseases. Its primary characteristic is the subendothelial deposition of lipids, leading to the formation of atherosclerotic plaques. Plaque stability directly dictates the risk of acute cardiovascular events, with intraplaque angiogenesis exerting a paradoxical dual regulatory role. Hypoxia-driven neovascularization enhances plaque stability through improved oxygen perfusion and facilitated macrophage migration to the necrotic core, potentiating clearance of lipids and necrotic debris (1). Conversely, structurally compromised neovessels exhibit impaired integrity and heightened permeability, enabling erythrocyte extravasation and inflammatory cell infiltration that escalate risks of intraplaque hemorrhage and rupture (2). Structurally compromised neovessels result in impaired vascular integrity and heightened permeability, facilitating erythrocyte extravasation and inflammatory cell infiltration that substantially elevate risks of intraplaque hemorrhage and rupture. Consequently, this precarious equilibrium between pathological injury and compensatory repair governs the phenotypic destiny of atherosclerotic plaques.

Intraplaque angiogenesis in AS represents a complex pathophysiological process involving multifaceted cellular and mechanistic interactions. During early atherogenesis, angiogenesis functions as a compensatory response to intraplaque hypoxia and heightened metabolic demands. Glycolysis not only furnishes essential energy for this process but also directly potentiates endothelial cells proliferation and migration, thereby inducing vascular sprouting (3). Simultaneously, mast cells engage in microvascular network assembly through endothelial crosstalk, releasing pro-angiogenic factors including vascular endothelial growth factor (VEGF); mast cells-derived inflammatory cytokines further amplify VEGF expression, provisionally maintaining plaque structural integrity (4, 5). Vascular smooth muscle cells (VSMCs) augment VEGF secretion via erythrophagocytosis (6) and interact with pericytes through phenotypic switching (7), synergistically driving neovessel maturation. This integrated machinery orchestrates intraplaque angiogenesis. Paradoxically, such compensatory neovascularization may transform into a pivotal pathological driver of atherosclerotic progression. Structurally aberrant neovessels exhibit heightened fragility and permeability, predisposing to hemorrhage-prone plaque transformation (8). Extravasated erythrocytes and blood components exacerbate local inflammation, establishing a vicious cycle wherein inflammatory stimuli fuel pathological angiogenesis, which in turn recruits additional inflammatory infiltrates. Clinically, this angiogenic-inflammatory synergy manifests most detrimentally in high-risk cohorts, where it critically compromises the fibrous cap integrity (9). Consequently, while angiogenesis plays a crucial protective role in early plaque remodeling, persistent dysregulated neovascularization ultimately exacerbates plaque vulnerability and rupture risk.

Precision modulation of angiogenesis to stabilize atherosclerotic plaques represents a pivotal frontier in current research. Clinical interventions face intrinsic therapeutic limitations: while high-intensity statin therapy remains the cornerstone of AS treatment, it only incompletely attenuates VEGF-mediated pathological neovascularization. Antiplatelet agents reduce platelet-derived exosome release by blocking the P2Y12 receptor, yet they fail to repair the already established leaky vascular networks. Consequently, elucidating the dualistic nature, protective yet disruptive, of intraplaque angiogenesis will establish the mechanistic foundation for developing both plaque vulnerability prediction models and targeted disease-modifying therapeutics, which will ultimately stabilize vulnerable plaques.

## 2 As angiogenesis-plaque stability paradox

Angiogenesis, the formation of new blood vessels from pre-existing vasculature, constitutes an essential process for tissue development and repair (2). Within AS plaques, angiogenesis can serve as a therapeutic tool promoting endothelial layer repair and plaque stabilization, while conversely representing a critical pathological process that drives plaque progression, induces intraplaque hemorrhage, and triggers plaque rupture (10). These functionally divergent plaque neovessels exhibit spatial heterogeneity shaped by local biomechanical forces, where positional architecture dictates plaque fate. This dual-capacity to generate diametrically opposing outcomes establishes the “angiogenesis-plaque stability paradox” concept in AS.

### 2.1 Angiogenesis stabilizes and repairs plaques

Angiogenesis plays a critical role in the metabolic activity of plaques. In AS, arterial wall thickening and inflammatory responses mutually reinforce each other, collectively driving plaque formation. Plaque accumulation reduces oxygen supply, while inflammation increases oxygen consumption, creating a hypoxic microenvironment within the plaque (10). The formation of new blood vessels mitigates the imbalance between oxygen supply and demand, enhancing the survival and metabolic activity of cells within plaques. Hypoxia and increased metabolic demand within plaques drive new vessel formation, which underscores the critical role of angiogenesis in supplying both oxygen and nutrients (9, 11). Meanwhile, neovessels also facilitate the transport of low-density lipoprotein (LDL) and the clearance of harmful substances (12), ameliorate lipid retention and inflammatory burden to decelerate plaque pathogenesis. This underscores the proactive role of angiogenesis in preserving plaque homeostasis and facilitating repair processes. However, the inherent fragility of intraplaque neovessels predisposes them to disruption, triggering hemorrhage and exacerbated inflammation that ultimately compromise plaque stability.

### 2.2 Vulnerability of angiogenesis and its impact on plaque rupture

**Figure 1:**
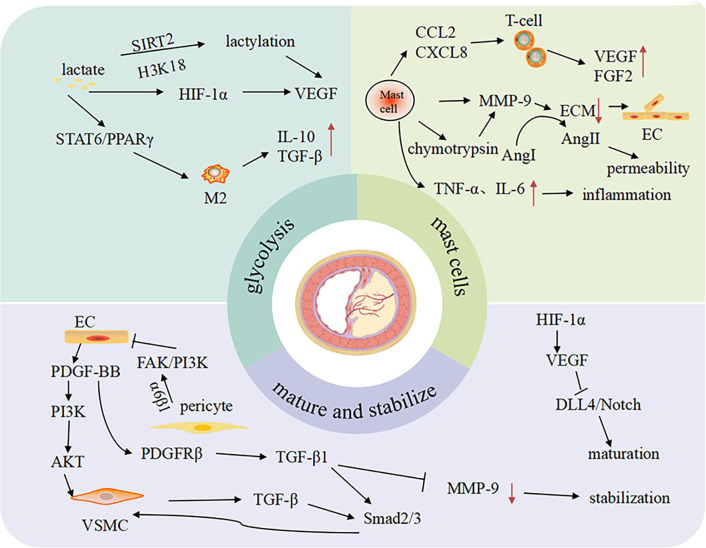
The related mechanisms of “angiogenesis-plaque stability paradox”.

Neovascular fragility constitutes the core pathological basis for plaque rupture. Compared to physiological vessels, the pathological neovascularization within the plaques exhibits disordered branching patterns, aberrant luminal dilation, and deficiency in endothelial junctional proteins. These structural defects heighten vascular permeability, creating pathological conduits for lipid infiltration, erythrocyte extravasation, and inflammatory cell migration into the plaque core (9). Infiltrating immune cells subsequently amplify local inflammation and oxidative stress, driving necrotic core expansion and escalating rupture risk. Concurrently, insufficient pericyte or VSMCs coverage compromises mechanical stability, predisposing neovessels to disruptive hemorrhage. Intraplaque hemorrhage not only perpetuates the inflammatory vicious cycle but also induces atypical ferroptosis (13, 14), further destabilizing plaque structural integrity. Therefore, mechanistic dissection of the “angiogenesis-plaque stability paradox” (Figure 1) will inform therapeutic strategies targeting neovascular stabilization to disrupt this self-amplifying pathological cascade.

## 3 The related mechanisms of the angiogenesis-plaque stability paradox

The angiogenesis-plaque stability paradox involves intricate mechanisms encompassing metabolic reprogramming, inflammatory dysregulation, impaired vascular maturation, and interpathway cross-talk. These regulatory networks differentially determine neovessel structure and function, thereby directly modulating the dynamic equilibrium between plaque stabilization and rupture predisposition (Table 1).

**Table 1:** Summary of studies on atherosclerosis plaque stability and angiogenesis paradox.

Mechanism of action	Experimental model	Central finding	Reference
Inhibition of endothelial glycolysis	ApoE^−/−^ PFKFB3 ECKO	Increasing M2 macrophage polarization	(3)
Upregulating PKM2-dependent glycolysis	ox-LDL-treated VSMCs	Upregulating PKM2-dependent glycolysis	(16)
TRAP1 increases aerobic glycolysis	ApoE^−/−^ KO Trap1 SMCKO	Increasing lactate-dependent H4K12la via HDAC3	(17)
PKM2 K305 crotonylation facilitates glycolysis	PDGF-BB-induced synthetic VSMCs	Enhancing PKM2 dimeric form	(18)
KLF4 enhances glycolysis	SMCs stimulated with TMAO or PDGF-BB	Upregulating PFKFB3 expression	(19)
NMAAP1 promotes glycolysis and lactate release	NMAAP1-CKO	Promoting M1 macrophage polarization	(21)
Upregulated glycolysis promotes H3K9 lactylation	VEGF-stimulated endothelial cells	Regulating angiogenesis through a feedback loop between H3K9la and HDAC2	(24)
Vascular remodelling	Sirt2 knockout mice	SIRT2 as a potential target for vascular rejuvenation	(27)
Glycolysis-mediated macrophage polarization	ox-LDL-induced RAW 264.7 macrophages	Upregulation of KLF2 alleviates atherosclerosis	(30)
Inhibition of inducible glycolysis reduces inflammation	PFK158-treated Ldlr^−/−^ mice	Inhibition of PFKFB3 stabilizes plaques	(31)
Mast cells activation	Acalabrutinib-treated Ldlr^−/−^ mice	Inhibiting IgE-mediated mast cell activation by Acalabrutinib	(34)
Mast cells activation	ApoE^−/−^ mice	Reducing mast cell number and activation	(35)
Mast cells release pro-inflammatory mediators	Ldlr^−/−^ mice	Mast cell stabilization leading to reduced inflammation	(36)
Mast cells activation	AGE-LDL-stimulated mast cells	Inhibition of ERK1/2 and NF-κB pathways	(39)
Mast cells activation	MC-specific inducible Srf knockout mice	Recruitment via PDGFB-PDGFRB signaling signaling	(45)
Phenotypic switching of SMCs	SMC-lineage tracing mice	Blocking transition to SEM cells, reducing atherosclerotic burden, and stabilizing fibrous cap	(46)
Macrophage-like VSMCs	STAT3 conditional knockout in VSMCs	Inducing macrophage-like phenotype via STAT3β	(48)
sPDGFRβ maintains pericyte quiescence	Acute hypoxia model	Dysregulated PDGFRβ leading to pericyte defects	(49)
Inhibits SMC-to-macrophage transition	ANGPTL4-injected Apoe^−/−^ mice	Reducing plaque size and inflammation by ANGPTL4	(52)
Pericyte contraction	Transient middle cerebral artery occlusion(tMCAO) model	Reducing pericyte contraction through inhibition of RHOA/ROCK1	(53)
Maturation of nascent vessels	Hypercholesterolaemic ApoE3*Leiden mice	Controlling neovessel maturation and inhibiting intraplaque hemorrhage	(54)
Maturation of nascent vessels	LPS-treated RAW264.7 macrophages	Suppressing M1 polarization through the TLR4-NFκB/MAPK pathway	(55)
Neovessel maturation	ApoE3*Leiden mice with vein graft	Inhibiting plaque formation by increasing neovessel maturation via PCmAb	(56)
Neovessel maturation	ApoE^−/−^ mice	Restraint of VEGF/VEGFR-2 signaling	(57)
Vascular mature and homeostasis	High glucose-induced pericyte injury	Reducing pericyte injury through circ_0001186 knockdown	(58)
Pericyte dysfunction	ox-LDL-induced pericyte dysfunction	Improvement of plaque stability through TGF-β1/Smad2/3 signaling	(60)
HIF-1α-Apelin/APJ and Ang-1/Tie signal pathways	ApoE^−/−^ mice	Reduction of plaque area, suppression of neovascularization, and promotion of maturation by SMYA	(63)
Apelin/APJ regulates EPC proliferation	Hypoxia treatment of EPCs	Role in EPC proliferation regulation	(64)
Apelin/APJ mediates monocyte adhesion	CRISPR-mediated sec62-KO in ECs	Regulating monocyte adhesion to endothelial cells	(67)
Apelin induces SMC phenotypic transition	Apelin-induced SMC transition model	Apelin-mediated phenotypic transition in intimal smooth muscle cells	(68)

### 3.1 The glycolysis-lactate-lactylation modification axis

#### 3.1.1 Regulatory mechanisms of glycolytic metabolism in plaque cells

Glycolysis serves as the primary energy source for vascular cells within AS plaques. Its unique dual-mode regulation—balancing oxygen dependence with hypoxia adaptation—drives AS progression and plaque destabilization by mediating endothelial dysfunction, synthetic phenotype switching in VSMCs, and inflammatory polarization of macrophages. In endothelial cells, glycolysis rapidly generates energy to accommodate environmental fluctuations, while its metabolic byproduct lactate concurrently influences cellular survival. However, hyperactivated glycolysis induces aberrant endothelial proliferation, thereby accelerating atherosclerotic progression and intraplaque pathological angiogenesis (3, 15). The proliferation, migration, and senescence of VSMCs are critical drivers in the development of AS, with glycolytic reprogramming constituting the core driver. Glycolysis is amplified via KLF4-driven post-translational modifications of 6-phosphofructo-2-kinase/fructose-2,6-bisphosphatase 3 (PFKFB3) and pyruvate kinase M2 isoform (PKM2). This metabolic shift drives VSMCs transition to a synthetic phenotype, exacerbating proliferation, migration, and senescence processes, thereby promoting vascular remodeling and plaque destabilization (16–19). Among macrophages, enhanced glycolysis represents a critical metabolic signature of M1 polarization, enabling adaptation to hypoxic inflammatory microenvironments while sustaining immune functionality (20). Studies demonstrate that bacillus calmette guerin stimulation potentiates glycolytic flux via the novel macrophage activation-associated protein 1, thereby driving macrophage polarization toward the M1 phenotype through amplified lactate production (21).

Operating as the master metabolic regulator of plaque vulnerability, glycolytic rewiring destabilizes atherosclerotic lesions via three synergistic axes: Pathological angiogenesis driven by aberrant EC hyperproliferation, fibrous cap disintegration via VSMC synthetic switching-mediated matrix degradation, and inflammasome propagation fueled by M1 macrophage polarization (22). This tri-directional disruption of vascular, stromal, and inflammatory integrity designates glycolytic metabolism as a clinically actionable target with compelling translational tractability.

#### 3.1.2 Lactate and lactylation-mediated modulation of angiogenesis

Lactate, the terminal metabolite of glycolysis, orchestrates angiogenesis through integrated metabolic control and epigenetic lactylation (23). Metabolically, lactate stabilizes hypoxia-inducible factor-1α (HIF-1α) to potentiate its transcriptional activity, thereby inducing expression of pro-angiogenic genes including VEGF (24). Concurrently, it drives macrophage polarization toward the M2 phenotype via the signal transducer and activator of transcription6 (STAT6)/peroxisome proliferator-activated receptor *γ* (PPARγ) signaling axis, enhancing secretion of pro-angiogenic factors IL-10 and TGF-β (25). In the field of epigenetics, lactate-derived lysine lactylation regulates chromatin openness and activates pro-angiogenic gene transcription by targeting histone H3 at lysine 18 (H3K18la) (26). Further mechanistic studies reveal that lactate finely regulates the spatiotemporal activation patterns of angiogenesis-related signaling pathways via a Sirtuin2 (SIRT2)-mediated lactylation-deacetylation dynamic equilibrium network (27, 28). These findings break the traditional view of lactate as merely a metabolic waste product, revealing its dual roles as a signaling molecule and an epigenetic substrate (29). By intervening in the transcription and expression of angiogenesis-related genes, this paradigm provides novel strategies for vascular-targeted therapies within AS plaques and regenerative approaches for ischemic tissues. However, translating lactate-targeting basic research into clinical applications remains challenged by formidable translational barriers, where inadequate targeting precision and rapid systemic clearance constitute primary roadblocks. To overcome these hurdles, future efforts should pioneer advanced nano-delivery platforms—exemplified by catalase-loaded porous polylactic acid biomimetic nanoparticles—leveraging single-cell sequencing-guided membrane engineering for surface functionalization. This dual-targeting, multi-mechanism synergetic strategy aims to reprogram the vascular niche microenvironment, concurrently achieving dual therapeutic objectives: revascularization maturation in ischemic tissues and precision interception of pathological neovascularization in atherosclerotic plaques.

#### 3.1.3 Targeting the lactate-histone lactylation axis in AS

The aberrant activation of the glycolytic pathway is closely linked to lactate metabolic dysregulation, and their interplay plays a pivotal role in the pathogenesis of AS. Targeted inhibition of glycolysis significantly reduces intraplaque cellular proliferative activity and pro-inflammatory cytokine release. Glycolysis inhibitors effectively suppress macrophage polarization toward pro-inflammatory phenotypes, thereby delaying the initiation and progression of AS plaques (30, 31). Beyond its role in energy metabolism, lactate has emerged as a pivotal signaling molecule, particularly through lactylation-mediated epigenetic regulation, thereby unveiling novel dimensions in angiogenesis research. Experimental evidence (32, 33) confirms that modulating lactate dehydrogenase activity reshapes the expression profile of key angiogenic factors within tumor microenvironments. Given that pathological neovascularization constitutes a shared hallmark of AS and oncogenesis, this discovery provides critical mechanistic parallels for targeting aberrant vascular proliferation within AS plaques. Building upon these insights, current research prioritizes developing small-molecule compounds dually targeting lactate transporters and lactylation-modifying enzymes. This dual-pronged strategy aims to co-regulate metabolic lactate homeostasis and post-translational modification networks, thereby engineering precision therapeutic approaches for vascular remodeling, thus accelerating the translation of metabolic interventions from bench to bedside.

### 3.2 Mast cell-mediated inflammatory response

#### 3.2.1 Mechanisms underlying mast cell accumulation and activation in as plaques

Mast cell-mediated inflammation represents a critical pathogenic axis in AS progression and plaque rupture, a process initiated by their site-specific accumulation and activation within lesions. Bone marrow-derived mast cell precursors are recruited and mobilized to plaque microenvironments via inflammatory mediators, including MCP-1, interleukin-8 (IL-8), tumor necrosis factor-α (TNF-α), and interferon-γ (IFN-γ), where they differentiate into mature subsets (34). Under the synergistic control of HIF-1α and local inflammatory signals, mature mast cells exhibit enhanced chemotactic activity, leading to their selective enrichment in plaque shoulders and necrotic cores (5). Upon activation, mast cells orchestrate complex inflammatory cascades through degranulation: Histamine increases vascular permeability via H1 receptor-mediated endothelial gap formation, facilitating monocyte/macrophage infiltration; proteases (tryptase/chymase) directly degrade extracellular matrix (ECM) components and activate matrix metalloproteinases (MMPs), thus destabilizing fibrous cap integrity; IL-6 and TNF-α drive phenotypic switching of VSMCs toward the matrix-degrading syntheses. Furthermore, mast cells fuel late-stage plaque vulnerability through paracrine release of VEGF and fibroblast growth factor-2 (FGF-2), stimulating pathological intraplaque neovascularization that precipitates intraplaque hemorrhage and rupture (35). This cascade highlights the therapeutic nodes targeting mast cell infiltration, activation, and mediator release as promising strategies to decelerate AS progression and stabilize plaques.

#### 3.2.2 Mast cell-mediated pro-angiogenic mechanisms

In AS plaques, mast cells drive pathological angiogenesis and plaque destabilization through a dual mechanism. Firstly, upon activation, mast cell-derived MMP-9 degrades type IV collagen and gelatin in the ECM, thereby compromising the structural integrity of the vascular basement membrane. This degradation process creates spatial conditions conducive to endothelial cell migration and subsequent lumen formation. Moreover, mast cell-derived chymase potently activates the pro-MMP-9 zymogen into its catalytically active form and cleaves angiotensin I to generate angiotensin II, amplifying vascular permeability and inflammatory infiltration. In LDLR^−/−^ mouse models, mast cell activation markedly exacerbates aortic lesion area, and promotes intraplaque angiogenesis, concomitant with upregulated MMP-9 levels (36–38). Furthermore, mast cells establish chemokine gradients that recruit monocytes and T lymphocytes into plaques. These infiltrating immune cells subsequently release pro-angiogenic factors such as VEGF-A and FGF-2. Collectively these mechanisms indicate that mast cell stabilizers reduce plaque MMP-9 activity while suppressing VEGF receptor phosphorylation, which will unveil targets to inhibit pathological angiogenesis.

#### 3.2.3 Mast cells and plaque instability

Mast cells not only promote intraplaque angiogenesis but also critically contribute to plaque destabilization. Although they may exert immune surveillance functions in early AS, their hyperactivation ultimately leads to destructive consequences. Research demonstrates (39) that mast cell-specific secretion of matrix metalloproteinases (MMP-9, MMP-2) and pro-inflammatory cytokines (TNF-α, IL-6) degrades ECM components, compromising fibrous cap integrity. Simultaneously, these inflammatory mediators synergistically induce VSMCs apoptosis, leading to impaired fibrous cap repair capacity. This matrix metabolic imbalance and cellular dynamic dysregulation significantly increase the risk of plaque rupture, serving as the initiating trigger for acute coronary syndrome (ACS). Single-cell RNA sequencing detects mast cells in vulnerable plaques of ACS, with a significantly positive correlation observed between mast cells infiltration and MMP-9 expression levels within these plaques (40). Additionally, mast cell-derived mediators (e.g., histamine, tryptase) promote platelet aggregation and fibrin deposition by activating protease-activated receptor 2 on endothelial cells and upregulating P-selectin expression on platelets, thereby establishing a pro-thrombotic microenvironment. Both animal experiments and clinical pathological studies have confirmed that mast cell infiltration is significantly correlated with plaque rupture and subsequent thrombus formation (34, 41, 42). Future research should focus on: Decoding dynamic evolution of mast cell functional subsets in patient biopsies, constructing phase-specific maps correlating subsets with clinical stages of AS, providing frameworks for developing stage-specific precision therapies.

### 3.3 Neovascular maturation and stabilization

#### 3.3.1 VSMCs recruitment and vascular wall remodeling

VSMCs maintain vascular wall homeostasis by orchestrating vascular development, homeostasis maintenance, and pathological remodeling. In AS, VSMCs undergo phenotypic switching and migrate to the intima, forming a fibrous cap enriched with α-smooth muscle actin (α-SMA) and ECM. The secretion of collagens I/III and elastin significantly enhances plaque mechanical strength. Molecular mechanism studies demonstrate that VSMCs activate the Smad2/3 signaling pathway by releasing TGF-β, which upregulates tissue inhibitors of metalloproteinases expression, thereby suppressing AS plaque matrix degradation (43). Moreover, VSMC-endothelial cell crosstalk underpins vascular maturation and stability (44). These cells form a functional unit where endothelial-derived platelet-derived growth factor BB (PDGF-BB) induces VSMCs proliferation through the phosphatidylinositol 3-kinase (PI3K)/protein kinase B (AKT) pathway, while VSMC-secreted hepatocyte growth factor enhances endothelial barrier function via the mesenchymal-epithelial transition factor receptor (45). This bidirectional paracrine regulatory network plays a pivotal role in vascular injury repair. However, under pathological stimuli, VSMCs can adopt macrophage-like phenotypes, participating in vascular inflammation and upregulating adhesion molecules (e.g., ICAM-1, VCAM-1). This increases vascular permeability, recruiting inflammatory cells and lipids to expand the necrotic core (46–48). In summary, VSMCs sustain vascular stability through structural support, matrix remodeling, and cellular interactions. Yet their pathological transformation drives plaque destabilization. Identifying key molecular targets to steer VSMCs toward beneficial phenotypes represents a critical frontier for future AS therapeutics.

#### 3.3.2 Pericyte–ECM interplay in vascular remodeling

Pericytes and ECM components constitute a core functional unit maintaining vascular homeostasis through structure-function coupling. Their synergistic interactions operate at three hierarchical levels: Structural-signaling coordination for barrier integrity, pericytes specifically express platelet-derived growth factor receptor β (PDGFRβ) (49), which senses and responds to PDGF-BB signals within the matrix-microenvironment, enhancing endothelial tight junction protein expression to reinforce vascular barrier function (50). Furthermore, pericytes actively anchor to collagen IV and laminin networks via integrin α6β1 receptors. This engagement activates the focal adhesion kinase (FAK)/PI3K signaling pathway, converting mechanical support into anti-apoptotic chemical signals that collectively preserve microvascular integrity (51). Bidirectional regulation with VSMCs for structural stability, pericyte-derived TGF-β1 induces contractile phenotypes differentiation in VSMCs via Smad2/3 phosphorylation, while VSMC-secreted angiopoietin-like 4 reciprocally regulates pericyte migration (52). This dynamic crosstalk critically depends on a healthy matrix microenvironment, with its disruption being pivotal during atherosclerotic plaque progression. Pericyte depletion and matrix disruption driving plaque destabilization, pericyte loss elevates MMP-2/MMP-9 activity in the fibrous cap, triggering matrix degradation (53, 54). Degraded elastin fragments shift from stabilizing elements to pathogenic signals, activating macrophage inflammasomes via Toll-like receptor 4 (TLR4)/myeloid differentiation factor 88 (MyD88) pathway and exacerbating local inflammation (55). In ApoE3*Leiden mouse models (56), pericyte coverage positively correlates with matrix stability, highlighting their synergistic significance and potential as plaque stability biomarkers. The pericyte-matri*x* axis orchestrates functional synergy across mechanical support, signal transduction, and immunomodulation. Targeting this integrated system will open new avenues for vascular microenvironment remodeling therapies.

#### 3.3.3 Failed neovessel maturation induces plaque destabilization

Impaired neovessel maturation constitutes a pivotal mechanism driving the formation of pathologically fragile vasculature, thereby disrupting plaque stability (57, 58). This process involves multicellular dysregulation: Disrupted pericyte-endothelial cell communication, disruption of the PDGF-BB/PDGFRβ signaling axis compromises pericyte-endothelial coupling, leading to inadequate pericyte coverage and defective basement membrane development in neovessels (59). Studies confirm that AS plaque neovessels exhibit pathologically reduced pericyte coverage index, which demonstrates a significant inverse correlation with intraplaque hemorrhage (60). VSMC-driven ECM homeostatic imbalance, aberrant VSMC phenotypic switching induces critical downregulation of α-SMA expression, resulting in compromised vascular wall tension. Concurrently, suppression of the TGF-β/Smad signaling axis drives stoichiometric collapse of collagen/elastin homeostasis (61). Inflammatory cascades drive ECM hyper-catabolism, monocyte recruited into plaques and polarized into proinflammatory M1-like macrophages, establishing a self-sustaining inflammatory niche. This microenvironment activates the nuclear factor-κB (NF-κB) pathway, inducing pathological overexpression of matrix metalloproteinases (MMP-2, MMP-9). These proteases degrade basement membrane components (collagen type IV, laminin), ultimately increasing vascular permeability (62). These mechanisms collectively cause vascular structural defects, triggering erythrocyte extravasation. Hemoglobin breakdown products subsequently activate macrophages via CD163 receptors, establishing a self-perpetuating pro-inflammatory/pro-angiogenic cycle (56). Notably, HIF-1α-driven pathological angiogenesis exacerbates vascular leakage through VEGF/Notch signaling imbalance, whereas blockade of Delta-like ligand 4 (DLL4) enhances neovessel maturation. Therapeutic interventions targeting pericyte recruitment enhancement, ECM metabolism modulation, and suppression of inflammation-hypoxia synergy demonstrate significant potential for stabilizing vulnerable plaques.

## 4 Regulatory mechanisms of intraplaque angiogenesis signaling networks

### 4.1 HIF-1α and angiopoietin-like protein (Apelin)/APJ signaling pathways

The HIF-1α/Apelin/APJ signaling axis exerts dualistic roles in AS, functioning as both a protective mediator and pathological driver. As the master transcriptional regulator of hypoxia-responsive genes, HIF-1α stabilizes and activates under intraplaque hypoxia and oxidative stress, driving transcriptional upregulation of Apelin and its G protein-coupled receptor APJ (63). Activated Apelin/APJ signaling promotes endothelial cell proliferation, migration, and neovascularization through the PI3K/AKT/mTORC1 axis to alleviate tissue hypoxia (64, 65), while concurrently activating Nrf2 through the CaMKK/AMPK/GSK3β pathway, thereby upregulating antioxidant enzymes (e.g., SOD, HO-1) to protect endothelium from oxidative damage (65, 66). Paradoxically, this axis accelerates AS progression by: Inducing NF-κB/JNK-mediated inflammation, upregulating ICAM-1, VCAM-1, and MCP-1 to exacerbate endothelial inflammation/permeability (67). Triggering nuclear translocation of calcium-binding protein A4, which induces synthetic phenotype transition in VSMCs, directly fueling plaque advancement (68). This functional duality implies that systemic pathway inhibition may compromise physiological repair mechanisms, necessitating future cell-type-specific therapeutic strategies. Precision approaches should combine microvascular ultrasonography with biomarker profiling for patient stratification, establishing distinct therapeutic windows, including pro-angiogenic intervention for hypoxia-dominant phases and anti-inflammatory targeting for inflammation-dominant stages.

### 4.2 The angiopoietin1/2 (Ang1/Ang2) and tie receptor signaling axis

The Ang-Tie signaling axis functions as a master regulatory hub for endothelial homeostasis by orchestrating: Vascular quiescence status, microvascular permeability, barrier stabilization, and controlled angiogenic progression (69). Ang1 and Ang2 act as agonistic and antagonistic ligands, respectively, for the endothelial tyrosine kinase receptor Tie-2. Ang1 promotes vascular structural stabilization, while Ang2 disrupts junctional integrity between endothelial cells and pericytes, increases vascular permeability, and antagonizes Ang1-mediated stabilization. Within AS plaques, Ang1 remodels neovasculature to reduce permeability, maintaining vascular maturation and stability.

Conversely, Ang2 orchestrates basement membrane remodeling and drives endothelial cell migration via MMP-2 proteolytic activation, culminating in pathological angiogenesis in the AS niche. In vulnerable plaques, Ang1 and Ang2 expression exhibits a pronounced imbalance dominated by Ang2. This pathological imbalance directly instigates microvascular fragility and potentiates plaque rupture vulnerability (70). Furthermore, Ang-2 modulates vascular growth, maturation and regression in tumors and vasculopathies through synergistic cooperation with pro-angiogenic factors including VEGF. Hence, elucidating the Ang1/Ang2 interplay with Tie receptors and their spatiotemporal dynamics within AS microenvironments establishes a framework for targeted intraplaque angiogenesis control and innovative therapeutic translation.

### 4.3 Mitogen-activated protein kinase kinase (MEK)/extracellular regulated protein kinases (ERK) and PI3K/AKT

The MEK/ERK and PI3K/AKT signaling pathways constitute dual regulatory axes governing AS plaque evolution, orchestrating the maintenance and destabilization of plaque phenotype through divergent yet complementary mechanisms. Activation of the MEK/ERK signaling pathway primarily orchestrates the proliferation of VSMCs and the propagation of inflammatory responses (71). MEK inhibitors demonstrate significant plaque volume reduction coupled with amelioration of inflammatory burden (72), underscoring the pivotal role of MEK/ERK pathway inhibition in plaque stabilization. In stark contrast, the PI3K/AKT pathway potentiates pathological angiogenesis by enhancing endothelial cell survival and migratory capacity. This process is further orchestrated through HIF-1α-mediated transcriptional control, resulting in aberrant microvascular networks at the base of AS plaques. These fragile neovessels serve as primary triggers for intraplaque hemorrhage and rupture (73). Critically, complex crosstalk exists between the MEK/ERK and PI3K/AKT pathways. Upon MEK/ERK inhibition, compensatory PI3K/AKT activation occurs via signaling nodes such as mTORC2. Conversely, PI3K/AKT blockade potentiates feedback-driven ERK hyperphosphorylation. Crucially, this reciprocal escape circuitry substantially compromises monotherapeutic efficacy. From a systems biology perspective, dual-targeting strategies, which coordinately suppress the “proliferation-inflammation-angiogenesis” pathological triad while blocking compensatory escape routes, establishing a transformative paradigm to overcome current therapeutic bottlenecks in AS.

## 5 Conclusion

The “Angiogenesis-Plaque Stability Paradox” illuminates the intricate relationship between intraplaque angiogenesis and plaque stability, a seemingly contradictory yet profoundly interconnected dynamic. While conventional perspectives predominantly emphasize the destabilizing role of angiogenesis in AS pathogenesis and progression, they often overlook its reparative function in maintaining plaque integrity. Modern research, however, reveals their deep pathophysiological interdependence. The glycolysis-lactate-lactylation axis and mast cell-mediated inflammatory cascades provide novel insights into metabolic reprogramming within the plaque microenvironment. Crucially, the maturity and stabilization mechanisms of neovessels demonstrate that vascular quality, not merely quantity, serves as the key determinant of its functional consequences. These advances collectively construct a multidimensional mechanistic framework for the paradox, while the regulatory circuitry governing plaque angiogenesis presents actionable therapeutic targets to resolve this duality.

In summary, by investigating shared pathological mechanisms and regulatory signaling circuits linking intraplaque angiogenesis to plaque stability, we reveal a complex paradox: Angiogenesis exerts beneficial effects in physiological repair contexts yet accelerates plaque destabilization under pathological conditions. The prevention and treatment research for AS and plaque rupture should prioritize precision discrimination and targeted modulation of pathological vs. protective intraplaque angiogenesis, mandating integrated consideration of critical determinants including therapeutic timing and drug specificity. Future advancements could leverage deep learning algorithms constructed upon optical coherence tomography angiography (OCTA) features to dynamically assess plaque stability through quantification of neovascular morphological parameters (including vessel density, branching complexity, and mural integrity) alongside spatial distribution patterns. Furthermore, wearable biosensors enabling real-time monitoring of angiogenic signatures may be developed, integrated with machine learning frameworks to establish alert systems with high predictive efficacy. Therapeutically, nanotechnology-based delivery platforms for multicomponent botanical formulations can be engineered to achieve multidimensional modulation of the atherosclerotic plaque microenvironment; this strategy integrates the inherent multicomponent synergy of Traditional Chinese Medicine with the spatiotemporal targeting advantages of nanomedicine, thereby concurrently regulating pathological angiogenesis while preserving essential reparative neovascularization to resolve the “angiogenesis-plaque stability paradox”. Consequently, the convergence of artificial intelligence-aided vascular imaging analytics and multitargeted precision control of the plaque microenvironment will furnish innovative solutions for enhancing plaque stabilization, simultaneously pioneering novel pathways within integrative Chinese-Western medical paradigms for atherosclerosis management.
